# Delay-aware distributed program caching for IoT-edge networks

**DOI:** 10.1371/journal.pone.0270183

**Published:** 2022-07-19

**Authors:** Chang Kyung Kim, TaeYoung Kim, SuKyoung Lee, Seungkyun Lee, Anna Cho, Mun-Suk Kim

**Affiliations:** 1 Yonsei University, Seoul, Republic of Korea; 2 Sejong University, Seoul, Republic of Korea; Khon Kaen University, THAILAND

## Abstract

Edge computing is a novel network architecture that is in proximity to the end devices in an Internet of Things (IoT). As the IoT becoming a major factor in our daily life, provisioning a low response time of the services to IoT users through edge computing is an important problem. Caching necessary program data for the task in an edge node effectively reduces the response time of the computation task. However, due to the increase of IoT users and devices, it is noteworthy that limited-resource edge nodes would receive a number of tasks, having a heavy burden of processing the requests. Therefore, the limited resource and caching space at cloudlet need the careful design of the caching algorithm to utilize the space of multiple edge nodes and relieve the burden of computations. In this paper, we propose a cooperative program caching system that makes different edge nodes cooperatively store program data and cache the replicas of the data requested frequently to handle a number of requests from IoT users. In particular, we develop a cooperative caching algorithm that caches the appropriate number of data replicas depending on the number of requests on each cloudlet and the popularity of the data to minimize the response time. The simulation results show that the proposed cooperative caching algorithm can effectively reduce the response time for IoT users compared to other existing algorithms.

## 1 Introduction

The realization of Internet-of-Things (IoT) envisions ubiquitous connectivity among billions of smart devices over the future Internet. The IoT is being assimilated into human lives from healthcare, smart homes, intelligent transportation, and industrial manufacturing to smart cities [1, 2]. However, a proliferation of modern wireless applications demanding high-performance computation and the limited computing capabilities of IoT devices bring great challenges. To overcome these challenges, cloud computing has emerged for IoT applications with its rich computing capacity and storage. Although such a heavy computing task can be processed by the existing cloud paradigm, it is not suitable for the pervasive IoT system, due to long communication delay between IoT users and the cloud and this makes delay-sensitive applications impossible to implement (e.g. industrial IoT and Virtual Reality games) [1, 3]. To address this problem, processing the task at the network edge (referred to as ‘cloudlet’ in this paper) has been considered as a promising solution [1, 3, 4]. Specifically, IoT users can access the cloudlets through the low-latency wireless network, then they can request the computation task to the cloudlets or the cloud.

Computing a task not only requires the input data but also needs the program/library data. For instance, when smart glasses send an image of a human face as input for face recognition to the cloudlet, it has to load all the necessary program/libraries and user-specific code for execution (e.g. library for human recognition) [5, 6]. We refer to caching these data as *program data caching*. It may take seconds to load all necessary programs from the cloud. Hence, caching program data in the cloudlets can reduce the delay caused by loading the program from the cloud. That is, the popular program data is cached at the cloudlet and then, there is no need to load the program if the task is executed at the cloudlet which caches the program data. We refer to the caching input and output data of computation tasks as *content caching*. However, the input and output data are hardly reused in the IoT scenario having short life time of the input and output data [7].

Since cloudlets are deployed near IoT users, they can serve the task with lower task response time due to low transmission time. However, cloudlet has the limited caching storage so that cloudlet can only cache the limited number of the data. To overcome this problem [8–11] suggested the cooperative caching strategy, where the neighbor cloudlet can forward the requested data if the cloudlet, which first receives the request from IoT user, does not cache the requested data. Even if [8–11] suggested the cooperative caching algorithm, these works only focus on the content caching. In these works, they did not consider the program data caching. With an explosive increase of IoT users and the limited computing capacity of the cloudlet, it is noteworthy that a cloudlet would receive a large number of task requests, having a heavy burden of processing the requests [12]. Accordingly, if the multiple IoT users request numerous computation tasks requiring the same program data and there is only one cloudlet caches that program data, then the cloudlet needs to handle all of these massive task requests. In this case, the IoT user get the result with longer total response time due to the long waiting time of the requests at the cloudlet [13]. [14–16] consider the program data caching with the cooperative network, but they didn’t consider the waiting time which is caused by the numerous task requests from the IoT users. Therefore, in this paper, we employ a cooperative edge caching strategy that caches the replicas of popular program data, which are frequently used, into multiple cloudlets.

Specifically, we formulate the problem of selecting which cloudlet to cache each program data and replica for minimizing the total response time experienced by the IoT user. Since the service time at the cloudlet can be different depending on the computing workload of the task, we analyze the total response time of the task with the waiting time based on the M/G/1 queuing model for the problem formulation [17, 18]. Since the formulated problem is known to be NP-complete, we propose a heuristic caching algorithm to solve the total response time minimization problem. We also designed the distributed task protocol to provide the low response time in the cache-enabled IoT-edge networks by choosing the best destination cloudlet to process the incoming requests. We finally conducted simulations to evaluate the performance of our proposed algorithm in comparison to the existing cooperative caching strategy.

The main contributions of this paper are summarized as follows:

- We propose a cooperative edge caching strategy to handle a number of tasks from IoT users that are requested to be processed at a cloudlet. In the proposed strategy, program data are cached in different cloudlets and the task requests can be forwarded to the further cloudlet if the cloudlet near the IoT user does not cache the required program data. Moreover, replicas of the popular program data are cached in multiple cloudlets so that the tasks requiring the same data can be forwarded to the multiple cloudlets.
- We formulate the caching problem with the objective to minimize the total response time of the task. For the problem formulation, we not only investigate the transmission time of the task request in the cooperative network, but also model the waiting time of task request at the edge cloudlet as an M/G/1 queuing system. Then, the waiting time is incorporated into the total response time.
- We propose the heuristic algorithm to solve the problem formulation which is NP-complete. The proposed algorithm caches more than one same data into multiple cloudlets depending on the number of requests and popularity.
- We implement the proposed caching algorithm and evaluate its performance, compared to the existing cooperative caching strategy in terms of the total response time. It is demonstrated via the simulation results that our algorithm improves the total response time.

The remainder of this paper is organized as follows. In Section 2, we review some related work on caching for IoT system. Section 3 gives the system architecture considered for our caching algorithm. In Section 4 and 5, we formulate the caching problem and then provide the heuristic caching algorithm to solve the problem. We evaluate our algorithm and compare with other existing caching algorithms in Section 6. Finally, Section 7 concludes the paper with some discussions.

## 2 Related works

### 2.1 Edge computing in IoT system

Recently, IoT data analytics play an important role in predicting the status of our surroundings by processing and computing collected from a huge number of IoT users [1, 2]. Use cases benefiting such IoT data analytics include smart city video analytics, smart manufacturing, health care, etc [2, 3]. Nevertheless, many IoT devices have limited computing resources and storage to cache diverse data and locally execute the heavy computation task. Therefore, cloud computing has been adopted to provide IoT users with sufficient storage and processing capability through data centers in the cloud. That is, a massive amount of multi-modal data collected from numerous heterogeneous IoT users have to be transferred to the cloud for storage, computation, and decision-making. However, the cloud platform is usually located physically far from the IoT users causing slow response times. Users can no longer tolerate such slow responses of cloud-based computing when using IoT applications such as self-driving cars, and voice recognition. More recently, therefore, edge computing, which brings resources close to the end-users, has been recognized as a solution to reduce the response time for IoT applications [4, 19, 20]. [20] shows that the edge paradigm will reduce the latency and distribute network traffic in computation offloading. [19] propose the smart-edge algorithm for the joint optimization of computation, caching, and communication to address the disadvantages of traditional cloud computing in terms of communication delay and network load.

### 2.2 Data caching in IoT-edge system

There have been some efforts aimed at reducing the total response time of the computation task for supporting a number of IoT applications via edge caching [6, 8, 21, 22]. Recent work has applied content caching to IoT-edge systems to reduce redundant computations, delay, and energy consumption. Especially, an edge node caches the output data of the task [9] and intermediate task results that may use for future task executions [21]. While previous studies show that the computation content caching can minimize the total response time, the fundamental assumption on reusing the same task input, output, or intermediate data may not hold for many IoT applications [6].

Program data caching, on the other hand, caches the library/program data for processing IoT applications. Caching program at edge nodes is an effective way to relieve the burden of the backhaul network and to reduce the delay caused by initializing necessary program data or task migration due to the absence of the program [5, 6]. There have been some research related to task offloading and the program caching [6, 23, 24]. [23] jointly considering the access network selection and program placement to improve the response time for edge applications. [6] minimize the computation delay and energy consumption of the edge node by jointly consider the program data caching placement, computation offloading decisions, and system resource allocation. The previous works have shown that the program data caching can reduce the total response time of the task. In particular, it can reduce the initialization of an application such as loading required libraries and initializing user-specific code. However, previous works [6, 23] did not consider the cooperative edge to overcome the limited storage and computing resources caching less diverse data in the system.

### 2.3 Cooperative caching

Compared to the cloud, the storage capacity at the edge is relatively limited [25]. Unfortunately, as the volume of content keeps increasing, the caching capacity at the edge nodes gets more limited [9]. Thus, there have been some studies on cooperative caching to efficiently utilize the storage resources as well as to improve the hit ratio and data retrieval time in the edge-enabled networks [8, 26]. [26] suggests a dynamic cache replacement algorithm which considers in the tree network topology. The strategy calculates the node access frequency to achieve better cache placement performance. [8, 9] suggest a spatially caching strategy to construct cooperative networks of edge servers. For maximizing the storage efficiency of edge servers, each edge server does not cache the data which have been cached by its nearest edge server. Despite their meaningful works in content caching, the fundamental assumption on reusing task input and intermediate data may not suitable for many IoT applications. Similar to content caching, there have been also many cooperative systems in program caching to improve the storage efficiency and the computing delay [14, 21, 26]. [14] proposes an online edge service caching algorithm to reduce the traffic sent to the cloud by utilizing the cooperative features of base stations in mobile edge cloud. In [21], the author studies how to edge servers cooperatively process the data of multiple applications via the heuristic greedy method and caching strategy.

### 2.4 Motivation

The previous works [8, 9, 26] have shown that cooperative caching is effective in managing the network traffic and storage of edge servers in the edge-enabled computing system. In particular, cooperative caching can increase the diversity of cached data, leading to a high hit ratio. However, these previous works did not consider the waiting time of the user requests for data because they focused on *content caching*, where no computation is needed but only sending the requested data. Although [9, 14, 15] suggested the cooperative caching algorithms in *program data caching*, they have not considered the waiting time of the requests in the cloudlet due to numerous task requests from the IoT users.

It is addressed in [12, 27] that the long waiting time can be caused by numerous task requests from IoT users. Specifically, since the program is required to execute the task in the cloudlet, the waiting time on a cloudlet can be increased if there is only one cloudlet that caches the high demanded program data and multiple IoT users send the task requests that require the same program data. Yet, in these works [12, 27], when a cloudlet receives a request and does not cache the related program data, it can forward its request only to the cloudlet that is directly connected to itself. As a result, the options of the cloudlets to which the requests can be forwarded are limited.

Therefore, in this study, we propose the cooperative edge caching strategy to handle a large number of computation tasks requested by IoT users, requiring the same program data. Specifically, in the proposed caching strategy, replicas of the high demanded program data are cached into the multiple cloudlets based on the number of requests and data popularity. Hence, if the cloudlet that received the task requests from IoT users does not cache the required program data, then the requests can be forwarded to the multiple cloudlets, leading to lower response times of the tasks.

## 3 Cache-enabled IoT-edge system

In this section, we first introduce our network elements of the cache-enabled IoT-edge caching system. Then, we explain the caching protocol of the IoT-edge caching system.

### 3.1 Overall system architecture

**Fig 1:**
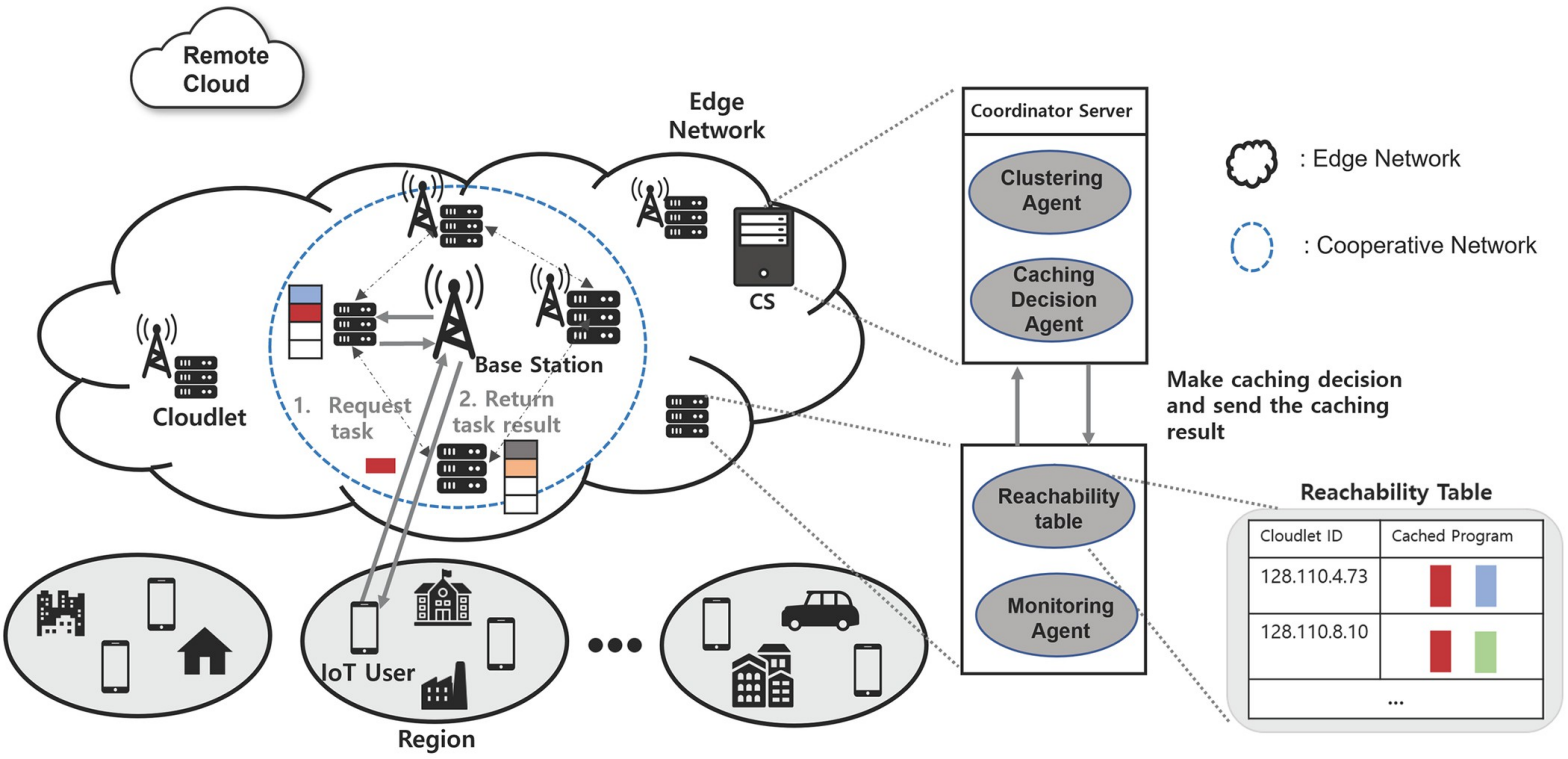
Cached-enabled IoT-edge system architecture.

The overall architecture of the IoT-Edge system is depicted in Fig 1. We consider a particular region that can be defined based on its features (e.g., residential district, business district) as IoT users will request the computing task related to these features [27, 28]. Each region consists of a Coordinator Server (CS), cloudlets, and IoT users. The role of each system element is defined as follows:

- **Coordinator Server**: CS is introduced to make a caching decision as a control server of our caching system. In each region, CS is installed as in [29, 30], while it may be replicated for different geographical regions. At the time when the CS is deployed, it retains the IP addresses of the cloudlets and RTT among the cloudlets in its region [27, 31].
  In the proposed system, CS mainly has the following two components:
  - Clustering Agent (CA): Since cloudlets are limited in the computing resources and caching space, CA forms clusters (referred to as ‘cooperative edge network’ in this paper) of cloudlets in edge networks [32]. Cloudlets in the same cooperative network can cache replicas of program data, so the tasks requiring the same program data can be distributed into multiple cloudlets. CS clusters the cloudlets when they are first deployed or later when a new cloudlet is additionally deployed in the region.
  - Caching Decision Agent (CDA): CDA is in charge of making a caching decision. CDA performs the caching decision when it receives the average number of task requests from the cloudlets of the region. After caching decision is finished, the CS sends the information of which cloudlet caches which program data as well as the estimated waiting time of each cloudlet to the cloudlets in its region. The waiting time refers to the time taken to finish executing task at the cloudlet. The process to calculate the waiting time of each cloudlet is explained in Section 5.
- **Cloudlet**: Cloudlet aims to bring computing capabilities and servers to the network edge and closer to users. Cloudlet is co-located with base station (BS) and deployed near IoT users to provide computing resources [28, 33]. In addition, cloudlets are usually deployed at some important sites, such as factories and schools [28, 32]. In these applications, it is necessary to optimize the task response delay of users. Recently, IoT applications such as augmented reality and natural language processing requires computation-intensive, resource-limited IoT users can send a task request to the cloudlets [28]. The main objective of using cloudlet is to process most of the data at the network edge and send less traffic toward the cloud, resulting in low data storage requirements and low total response time [33].
  If the cloudlet that receives a task from an IoT user does not cache the required program data for the task, the cloudlet can forward the task to another cloudlet in the same cooperative edge network. In the proposed system, each cloudlet has the following two components:
  - Reachability table: Each cloudlet contains the reachability table that has the waiting time and IP addresses of cloudlets in the same cooperative network, and the information of what program data each cloudlet has [27, 30]. The reachability table is updated after the caching decision is finished by the CS.
  - Monitoring Agent (MA): MA monitors the incoming task requests and stores the number of incoming requests for each program data during a certain period of time (e.g. a day) [34]. MA sends the stored information to CDA for caching decision.
- **IoT users**: IoT users are smart devices such as smart phones, wearable computing devices and self-driving cars [35]. To efficiently provision the real-time services for the applications, IoT users request the task with their collected data to one of near cloudlets.

### 3.2 Procedure of processing task requests

In this subsection, we describe how IoT user sends the task to the cloudlet and gets back a result of that task. The process of requesting a task and getting the result corresponding to the task work as follow:

1. When the IoT user wants to send a computation request, it first sends a request to the BS. BS finds the nearest cloudlet using the agent discovery process and forwards the request to the found cloudlet [36].
2. The cloudlet that first received the request from the IoT user works as a proxy cloudlet. If the proxy server contains the required data for the task, it executes the task and returns the result to the IoT user.
3. Otherwise, using the reachability table in the proxy cloudlet, the proxy cloudlet selects the cloudlet with the lowest waiting time among the cloudlets that has the required data for the task. Then, the selected cloudlet will execute the task. After the cloudlet finished executing the task, the cloudlet record what program data is used for the task using MA.
4. If none of the cloudlets can execute the task due to the absence of required program data, the IoT user directly sends the task to the cloud.

## 4 Analysis of total response time & problem formulation

### 4.1 System model

We denote by I the set of cloudlets in the region, *i* ∈ *I*. Let J and K be the sets of program data and IoT users, respectively. The program data *j*, *j* ∈ *J*, is required to process the computing tasks at the cloudlet.

We denote *s*_*j*_ as the size of the input data for the task requiring program *j*. For any program data *j*, *x*_*ij*_ is used to indicate whether the data is cached at the cloudlet *i*

$$\begin{array}{c} x_{ij}=\{\begin{array}{cc} 1, & \;\text{caches}\;\text{program}\;\text{data}\;j\\ 0, & \;\text{Otherwise}\; \end{array} \end{array}$$ (1)

We denote *n*_*ij*_ as the number of incoming requests requiring program data *j* to the cloudlet *i*. MA measures *n*_*ij*_ during a certain period of time, and sends the measured value to CDA in CS. Using the received *n*_*ij*_, CDA can obtain the popularity of each data *p*_*j*_ by calculating $p_{j}=\frac{\sum_{i\in I}n_{ij}}{\sum_{i\in I}\sum_{j\in J}n_{ij}}$. Then, using the Exponential Weighted Moving Average (EWMA), CDA obtains the average popularity ${\bar{p}}_{j}$ for data *j* at cloudlet *i* as [37]:

$$\begin{array}{c} {\bar{p}}_{j}=\left(1-\sigma \right){\bar{p}}_{j}^{*}+\sigma p_{j},\;\;\forall j\in J \end{array}$$ (2)

where *σ* ∈ [0, 1] is a weight and ${\bar{p}}_{j}^{*}$ is the average popularity obtained in the previous period.

Using the average popularity, ${\bar{p}}_{j}$ obtained from Eq 3 we can get the hit ratio of the system (denoted by *h*). The hit ratio is defined as the probability of finding the program required for the task in the cache of cloudlets. We then can express the hit ratio as:

$$\begin{array}{c} h=\sum_{j\in J}{\bar{p}}_{j}\left(1-\prod_{i\in I}\left(1-x_{ij}\right)\right) \end{array}$$ (3)

The above equation means the probability of finding of at least one cloudlet that caches the required program data in the IoT edge. The last part of Eq 3, ∏_*i* ∈ *I*_(1 − *x*_*ij*_) becomes zero if one of the cloudlets *i* ∈ *I* caches the program data *j*. The binary variable *x*_*ij*_ is decided by CDA in CS when it performs the caching decision. The details about the caching decision process will be explained in Section 5.2.

### 4.2 Transmission time between IoT user and cloudlet

If an IoT user transmits the task to an optimal cloudlet *i*, the transmission time between IoT users and the cloudlet *i*, *t*_*ij*_ can be expressed as:

$$\begin{array}{c} T_{hit}=\frac{s_{j}}{r_{i}} \end{array}$$ (4)

where *r*_*i*_ is the average transmission rate at the cloudlet *i* and *s*_*j*_ is the size of input data of the task that requires the program *j*. Since the size of the result is usually very small, the delay to receive the result is negligible [15, 30]. Each IoT user *k* will be allocated to a bandwidth *β* for transmitting its task. We denote *P*_*ik*_ and *c*_*ik*_ as the transmit power of and channel gain from IoT user *k* to *i*, respectively. Let *σ* is Gaussian noise, the transmission rate beteen *r*_*ik*_ achieved at IoT user can be written as $r_{i}=\beta log_{2}\left(1+\frac{P_{ik}\cdot {\left|c_{ik}\right|}^{2}}{\sigma^{2}+\sum_{k^{\prime }\in K\backslash \left\{k\right\}}P_{ik^{\prime }}\cdot {\left|c_{ik^{\prime }}\right|}^{2}}\right))$.

Then, we now can get the total transmission time including the cases of the task is executed in the cloudlet or the cloud, $T_{i}^{r}$:

$$\begin{array}{c} T_{i}^{r}=h\cdot T_{hit}+\left(1-h\right)T_{miss} \end{array}$$ (5)

where *T*_*miss*_ is the sum of total transmission time and execution time between IoT user and the cloud when the task is executed in the cloud.

### 4.3 Waiting time

When the task requests arrive at the cloudlet, each cloudlet is supposed to process one task at a time. A large number of task requests are sent to the cloudlet and so the tasks must be queued [12]. We assume that the computation task requests are generated by IoT users in the region according to a Poisson process. The time required to process the task depends on the computing workload of the task and the computing power of each cloudlet *i*. The tasks have different computing workloads following the distribution of the general service rate. Hence, we model the waiting time of requests at the cloudlet as an M/G/1 queuing system [17].

The multiple arrivals of task requests requiring program *j* can be distributed into multiple cloudlets in our considered architecture. Hence, we can express the expected arrival rate of task request requiring *j* to the cloudlet *i*, λ_*ij*_ as:

$$\begin{array}{c} \lambda_{ij}=\lambda \cdot \frac{x_{ij}\cdot {\bar{p}}_{j}}{Y_{j}} \end{array}$$ (6)

where *Y*_*j*_ is the number of cloudlets that contain the data *j* in a cooperative network which is given by *Y*_*j*_ = ∑_*i* ∈ *I*_ *x*_*ij*_. λ is the total arrival rate of task requests to the region (i.e λ = ∑_*i* ∈ *I*_∑_*j* ∈ *J*_λ_*ij*_). We then define the service time to process a task requiring program *j* at *i* as

$$\begin{array}{c} \frac{1}{\mu_{ij}}=\frac{c_{j}}{f_{i}} \end{array}$$ (7)

where *c*_*j*_ and *f*_*i*_ are the computational computing workload of request requiring program *j* and the computing power of cloudlet *i*, respectively.

Then we calculate the average number of task requests at *i* as:

$$\begin{array}{c} E[\frac{1}{\mu_{i}}]=\frac{\sum_{j\in J}\frac{\lambda_{ij}}{\mu_{ij}}}{\sum_{j\in J}\lambda_{ij}} \end{array}$$ (8)

We use Pollaczek-Khinchine formula to get the average queue length and use Little’s law to obtain the average waiting time of the request at the cloudlet *i* as:

$$\begin{array}{c} T_{i}^{w}=\frac{\sum_{j\in J}\lambda_{ij}E\left[{\left(\frac{1}{\mu_{i}}\right)}^{2}\right]}{2(1-\sum_{j\in J}\lambda_{ij}E\left[\frac{1}{\mu_{i}}\right])} \end{array}$$ (9)

#### 4.3.1 Minimization of total response time

Our main work is to make a caching decision for the cloudlets to reach the minimum total response time. The total response time, which is the interval between the moment when an IoT user sends a request and when it receives the result, is a sum of the transmission time and the waiting delay at the cloudlet.

By considering the waiting time at the cloudlet with the transmission time, the total response time of a task ${\hat{T}}_{i}^{r}$ can be rewritten as:

$$\begin{array}{c} {\hat{T}}_{i}^{r}=T_{i}^{r}+h\cdot T_{i}^{w} \end{array}$$ (10)

As RTTs (round-trip time) among cloudlets in the same cooperative network are set to be small in the clustering process, the time taken for finding the optimal cloudlet is neglected [35].

Denoting with *b*_*j*_ the size of program data, we formulate the caching problem to decide where to be cached (i.e., on which cloudlet) with the aim to minimize the total response time of the system as:

$$\begin{array}{cc} \min_{x_{ij}} & \sum_{i\in I}{\hat{T}}_{i}^{r} \end{array}$$ (11)

$$\text{s.t.}\;0<\sum_{j\in J}b_{j}x_{ij}\leq S_{i},x_{ij}\in 0,1$$ (12)

where the constraint Eq 13 indicates that a sum of cached data size cannot exceed the maximum capacity of the cloudlet *i*.

The main objective of 0–1 multiple knapsack problem is to maximize/minimize the function with respect to the decision variable that cannot exceed an upper bound. To prove that our problem is same as 0–1 multiple knapsack problem, we expand the proposed problem formulation as Eq 13 in below.

As shown in Eq 13, the problem objects to find the minimum value of total response time by deciding the value of binary indicator *x*_*ij*_ under the constraint of the caching capacity of the cloudlet. Hence, our minimization of the total response time problem is proven as a 0–1 multiple knapsack problem, which is proven as NP-complete.

$$\begin{array}{c} \begin{array}{c} \underset{x_{ij}}{\text{min}}\sum_{i\in I}{\hat{T}}_{i}^{r}=\text{min}\sum_{i\in I}(T_{miss}+\left(T_{hit}+T_{i}^{w}-T_{miss}\right)\sum_{j\in J}{\bar{p}}_{j}\prod_{i\in I}\left(1-x_{ij}\right)) \end{array} \end{array}$$ (13)

To address the proposed problem, we not only suggests the process of executing task at the optimal cloudlet but also introduce a distributed delay-aware caching algorithm in the following section.

## 5 Distributed delay-aware caching algorithm

In this section, we design the details of our clustering process and heuristic caching algorithm to minimize the total response time by caching appropriate number of same data into multiple cloudlets.

### 5.1 Clustering process

To prevent the request is sent to a far cloudlet which can cause an unexpected long transmission time, CS groups the cloudlets into clusters based on the RTT among them. For collecting RTT among cloudlets, CS sends lists of IP addresses to its cloudlets. Using the lists, cloudlets measure the RTT (e.g. ping mechanism using IP) among themselves. Then cloudlets return the measured RTT to the CS.

In the clustering algorithm, we introduce a control parameter, *t*_*cl*_ to limit the size of the cluster (i.e., the intra-cluster delay). The larger size of the cluster results in a higher *T*^*r*^, but a greater number of cloudlets. For the clustering process, CS performs the following process:

1. CS first finds a random cloudlet that is not included in any cluster, then makes this cloudlet as a start of a new cluster
2. CS finds the cloudlet *i* that is connected to the cluster by 1 hop. Cloudlet *i* can be included in *m* if it does not belong to any other cluster and if the *RTT* among cloudlet *i* and the other cloudlets in the cluster is less than *t*_*cl*_. Otherwise, the cloudlet *i* cannot be assigned to the cluster.
3. CS keeps expanding the cluster until there is no cloudlet that can be added to the cluster.

CS repeats the above process until there is no cloudlet that does not belong to any cluster. The clustering algorithm occurs at a moment when a new cloudlet is deployed in the system. After the clustering process is completed, CS starts caching data into its cluster using our proposed caching algorithm.

**Algorithm 1** Caching Algorithm

1: **Input**:

  *I*, *J*, *X*: matrix of *x*_*ij*_

2: **Initialize**:

  Sort program data set of *J* in descending order based on *p*_*j*_

3: **for each** *j* ∈ *J* **do**

4:  Obtain *i* that has the largest $S_{i}^{\prime }$

5:  *x*_*ij*_ ← 1

6: **end for**

7: Calculate *T*^*w*^ using Eq 10

8: $\text{min}{\hat{T}}^{w}=T^{w}$

9: **while** $S_{i}^{\prime }!=0$, in *i* ∈ *I* **do**

10:  **for each** *i* ∈ *I* **do**

11:   **for each** *j* ∈ *J* **do**

12:    **if** all *i* ∈ *I* is full **then**

13:     Terminate the Caching Algorithm

14:    **else if** $s_{j}>S_{i}^{\prime }$ **then**

15:     *x*_*ij*_ = 1

16:    **end if**

17:    Calculate ${\hat{T}}^{w}$ using Eq 10

18:    **if** ${\hat{T}}^{w}<\text{min}{\hat{T}}^{w}$ **then**

19:     $\text{min}\;{\hat{T}}^{w}={\hat{T}}^{w}$

20:    **else**

21:     *x*_*ij*_ = 0

22:    **end if**

23:   **end for**

24:  **end for**

25:  **if** $|T^{w}-\text{min}\;{\hat{T}}^{w}|<\theta$ **then**

26:   Terminate the Caching Algorithm

27:  **else**

28:   $T^{w}=\text{min}{\hat{T}}^{w}$

29:  **end if**

30: **end while**

### 5.2 Caching process

The 0–1 knapsack problem in Eq 12, can be solved using brute force, but the approach is not scalable as the number of data and cloudlets increases. Instead, the problem can be solved using the greedy algorithm by making a heuristic decision at each stage. Hence, we design the heuristic caching algorithm. Moreover, we introduce the control parameter *θ* which is an adjustable threshold to control the cache storage of cloudlets and the complexity of the algorithm. The algorithm is terminated when the reduction of waiting time during the caching process is less than *θ*. The larger value of *θ* may save the storage of cloudlets but caches less number of replicas may lead to more waiting time.

The CDA component in CS executes the following caching algorithm:

1. For each data *j* ∈ *J*, CS caches program data *j* into the cloudlet *i* which has the largest remaining storage ($S_{i}^{\prime }=S_{i}-\sum_{j=1}^{J}x_{ij}\cdot b_{j}$) (Line 3–6).
2. CS calculates the current waiting time *T*^*w*^ using Eq 10. (i.e. $T^{w}=\sum_{i\in I}T_{i}^{w}$) (Line 7).
3. For each data *j* ∈ *J* and each cloudlet *i* ∈ *I*, CS calculates the expected waiting time of the cooperative network ${\hat{T}}^{w}$ when additional data *j* is cached into the cloudlet *i*. Then CS selects *x*_*ij*_ that leads to lowest ${\hat{T}}^{w}$ when *x*_*ij*_ set to 1. (Denoted as $\text{min}{\hat{T}}^{w}$). However, The selected *x*_*ij*_ has to satisfy constraint $s_{j}>S_{i}^{\prime }$ (Line 10–24).
4. The algorithm is terminated if none of cloudlet can cache the data *j* due to full of storage (Line 12–13).
5. If the decrements of the total response time after caching selected data *j* into the cloudlet *i* is less than *θ* (i.e., $|T^{w}-\text{min}{\hat{T}}^{w}|<\theta$), caching process is terminated (Line 25–26). Then the algorithm is repeated from Step 3.

After the caching decision is finished, CDA sends the information of which cloudlet caches which program data and the estimated waiting time of each cloudlet to the cloudlets in the region. The information is updated into the reachability table of each cloudlet and cloudlets use the reachability table to select the cloudlet that executes the task.

## 6 Performance evaluation

### 6.1 Simulation settings

We consider the scenario in which 100 cloudlets are randomly distributed. The topology of the cloudlets is generated randomly in each experiment using a random graph generator. Then the RTTs among the cloudlets are uniformly distributed from 0.5 to 1.2 ms [27].

There are 50 types of program data, with the popularity of each program follows a Zipf-distribution with a shape parameter *α* = 7 and the popularity weight factor *σ* is set to 0.125 to avoid high fluctuations in the estimation [22, 38, 39]. The transmission power of IoT user is set from 0.2W to 0.3W [40, 41]. The bandwidth between cloudlets from users is 500 Mbps. Since the task request has the input data and the computing workload to be completed, we set the input data and output data size *s*_*j*_ as an average of 2 MB [42]. Since the waiting time at the cloudlet is designed as M/G/1 queuing, the computing workload of the request *c*_*j*_ follows uniform distributions in [75 × 10^6^, 200 × 10^6^] cycles [43].

We set the cloud’s computation capability is set to 2 times of the cloudlet [24]. The delay from users to the cloud is 0.3 s [36]. We initially set *t*_*cl*_ as 0.015 s [24, 27, 36], which constructs average of ten clusters in the entire simulation area. We simulate the algorithm by varying the arrival rate from λ_*i*_ = 1 to 10 (requests/s) for each cloudlet assuming the IoT user requests a task per second. The capacity of each cloudlet is set to range from 20—40% of the total size of unique data in the system so that the storage capacity of the (e.g. when *J* = 50, each cloudlet can store a maximum of 10 to 20 data) [12].

We compared our proposed algorithm to the other two methods. The first algorithm is the cooperative caching algorithm where the popular data is cached into the one cloudlet for each cooperative network (denoted as general cooperative caching (*CC*)). The second algorithm caches one replica for the popular data in the cooperative network (denoted as replica caching (*RC*)).

### 6.2 Simulation results

**Fig 2:**
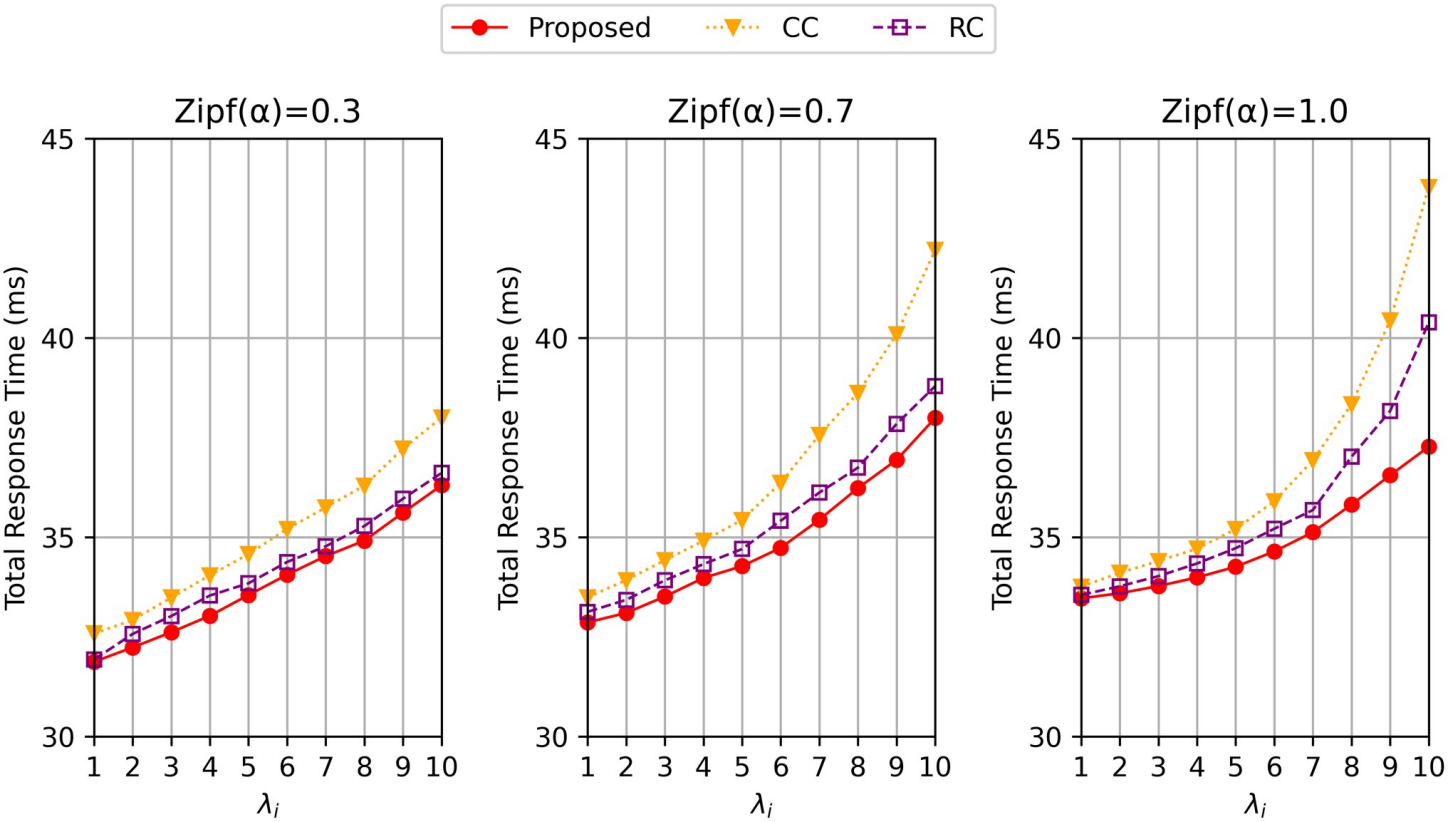
Total response time varying λ_*i*_ when the Zipf distribution parameter *α* = 0.3, 0.7 and 1.0.

In Fig 2, we show the effect of the arrival rate λ_*i*_ on the total response time for each algorithm with different Zipf parameters *α*: 0.3, 0.7, and 1.0. The proposed algorithm always outperforms the other two comparing algorithms for all the three values of *α* with respect to the total response time. The delay difference among the three algorithms gets larger when *α* is increased as shown in Fig 2, but the proposed algorithm relatively consistent because it adjusts the number of replicas depending on the popularity of the data. The smaller *α* implies the lower consistency of the user’s preference for the popular data. In other words, when *α* is high (e.g *α* = 1.0), the demand for tasks requests requiring popular data (i.e., the popularity is *p*_*j*_) is higher than when *α* is low. Accordingly, the waiting time at the cloudlets that contain the popular data gets higher. We thus can say that the proposed algorithm can achieve a relatively lower delay on various values of *α* by caching multiple replicas of the data that have higher popularity and arrival rate in the cluster.

**Fig 3:**
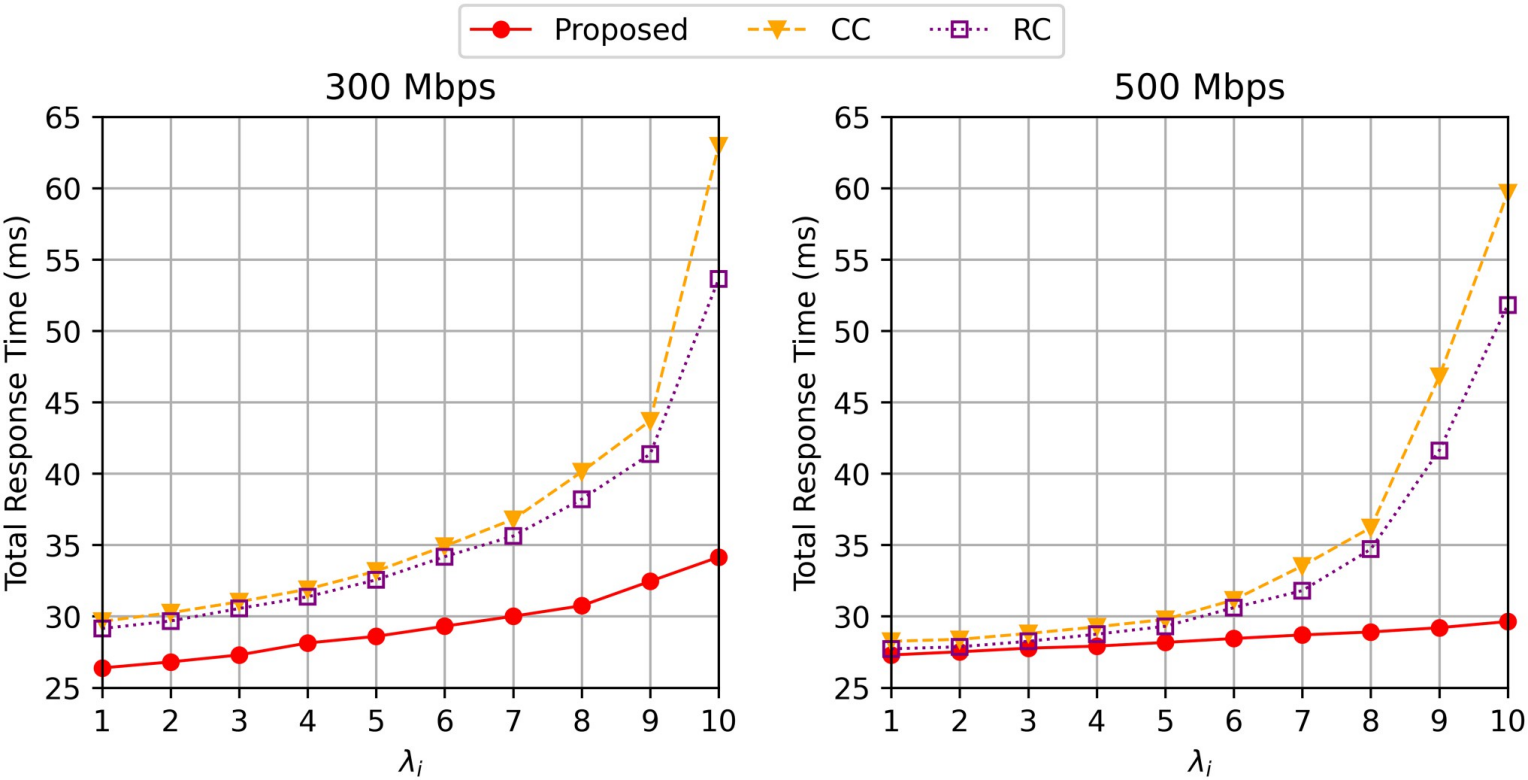
Total response time varying λ_*i*_ when the bandwidth *β* = 300 and 500 Mbps.

We plot Fig 3 describing the total response time with the different values of *β* between IoT users and cloudlet *i* to examine how transmission rate affects the total response time. The average delay of the three algorithms decreased with the higher transmission rate due to less transmission delay between the. IoT user and cloudlet. We also observe that all the three algorithms achieve higher response time with larger λ_*i*_ because the waiting time is increased due to the increase in the number of requests. As shown in Fig 3, *CC* and *RC* are less tolerable to the higher arrival rate compared to the proposed algorithm. Especially, the total response time of the two comparing methods are highly increased when the arrival rate (λ_*i*_) is bigger than 8. This is because *CC* and *RC* cache the one or two data for each program data in the cooperative network so that the requests requiring the same program data are concentrated to one or two cloudlets leading to the long waiting time at the cloudlet.

**Fig 4:**
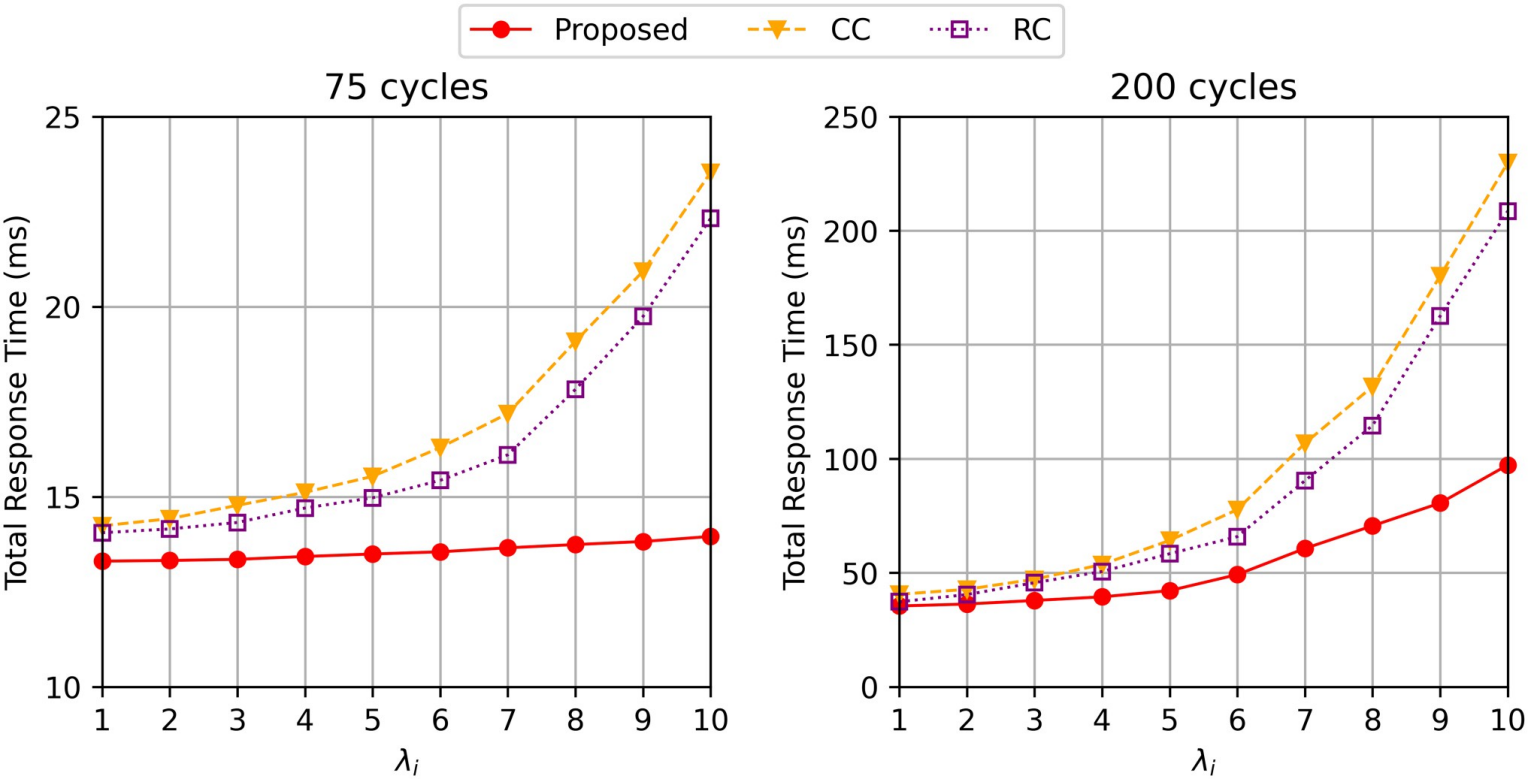
Total response time varying λ_*i*_ when the the computation workload of the task *c*_*j*_ = 75 and 200 cycles.

In Fig 4, we compare the total response time on the different computing workloads of the task. Our algorithm gets more effective when the task gets more complicated. We examined the three algorithms with different computational workloads of the task (*c*_*j*_): 75 × 10^6^ and 200 × 10^6^ cycles. We see from Fig 4 that when *c*_*j*_ is 75 × 10^6^ cycles, the proposed algorithm still shows a better total response time for all the different arrival rates. As shown in Fig 4, the total response time of *CC* and *RC* is highly increased from λ_*i*_ = 7. When *c*_*j*_ is increased to 200 × 10^6^ cycles, our proposed algorithms shows average of 28% and 19% a better total delay than *CC* and *RC* when λ_*i*_ is relatively low(i.e. λ_*i*_ ≤ 7). Then the total response time of the proposed algorithm significantly increases when λ_*i*_ gets larger than 7. As shown in Fig 4, while our algorithm still has a stable delay on a larger request intensity, the delays of *CC* and *RC* are significantly increased.

**Fig 5:**
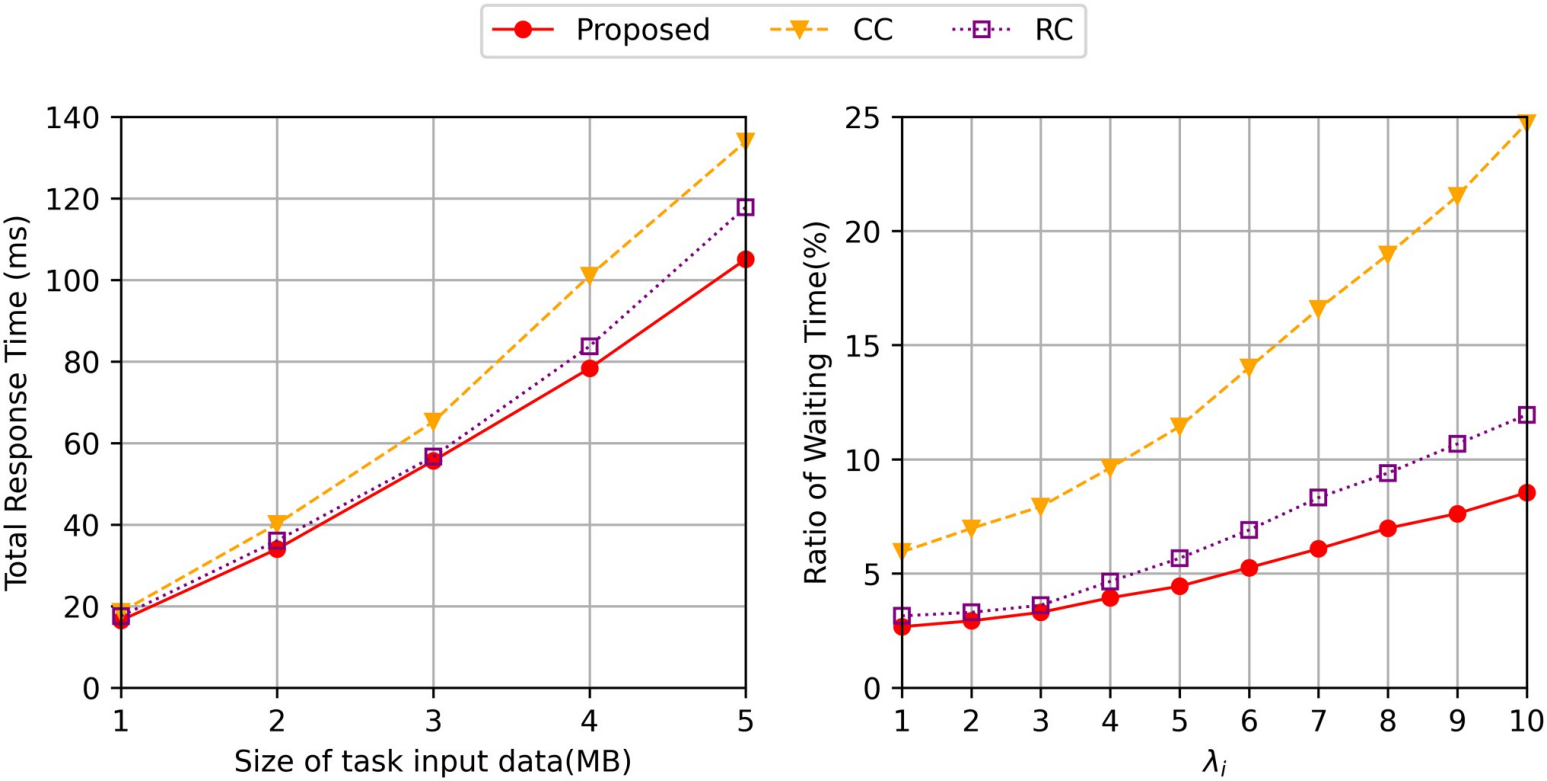
Total response time varying (left) the size of task data *s*_*j*_ and (right) the ratio of waiting time in total response time varying λ_*i*_.

In Fig 5 (Left), the graph shows the total response time depending on the size of input data *s*_*j*_. The proposed algorithm shows an average of 25% and 20% better total response time than the *CC* and *RC*. The control of waiting time is also important to minimize the total response time. Fig 5 (Right) shows the ratio of waiting time to total response time for three algorithms. As shown in Fig 5, the ratio of waiting time is increased as λ_*i*_ increases since more requests are held in each cloudlet. The ratio difference between the three algorithms shows that the waiting time has a large effect on the total response time. The waiting time of the proposed algorithm is relatively smaller than *CC* and *RC* also indicating that the waiting time can be reduced by caching replicas into multiple cloudlets and distributing the task requests.

**Fig 6:**
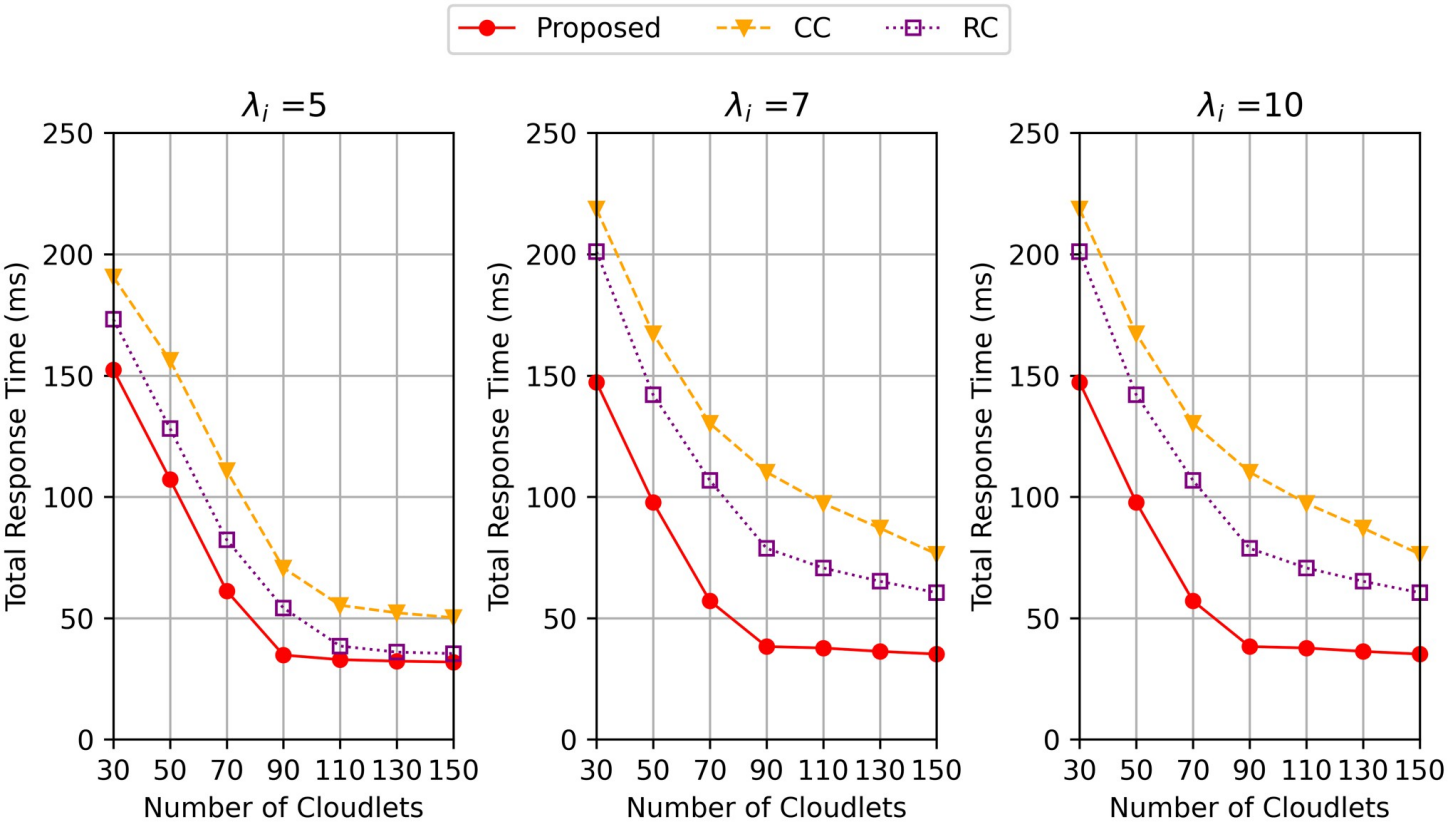
Total response time varying the number of cloudlets when λ_*i*_ = 5, 7 and 10.

Fig 6 plots the total response time versus the number of cloudlets increasing from 30 to 150 when λ_*i*_ = 5, 7 and 10 requests per second. We set the number of clusters as 10. We observe that as the number of cloudlets increases, the total response time decreases for all the three methods, while the proposed algorithm outperforms the other two methods. This is because the proposed algorithm can cache more program data in the cooperative network when there are more cloudlets, resulting in the task requests being distributed to more cloudlets. As shown in Fig 6 the differences in total response time between the proposed methods and the other two methods gets smaller as the number of cloudlets increases, but the delay differences between the proposed algorithms and the other two methods get larger when the arrival rate increases.

**Fig 7:**
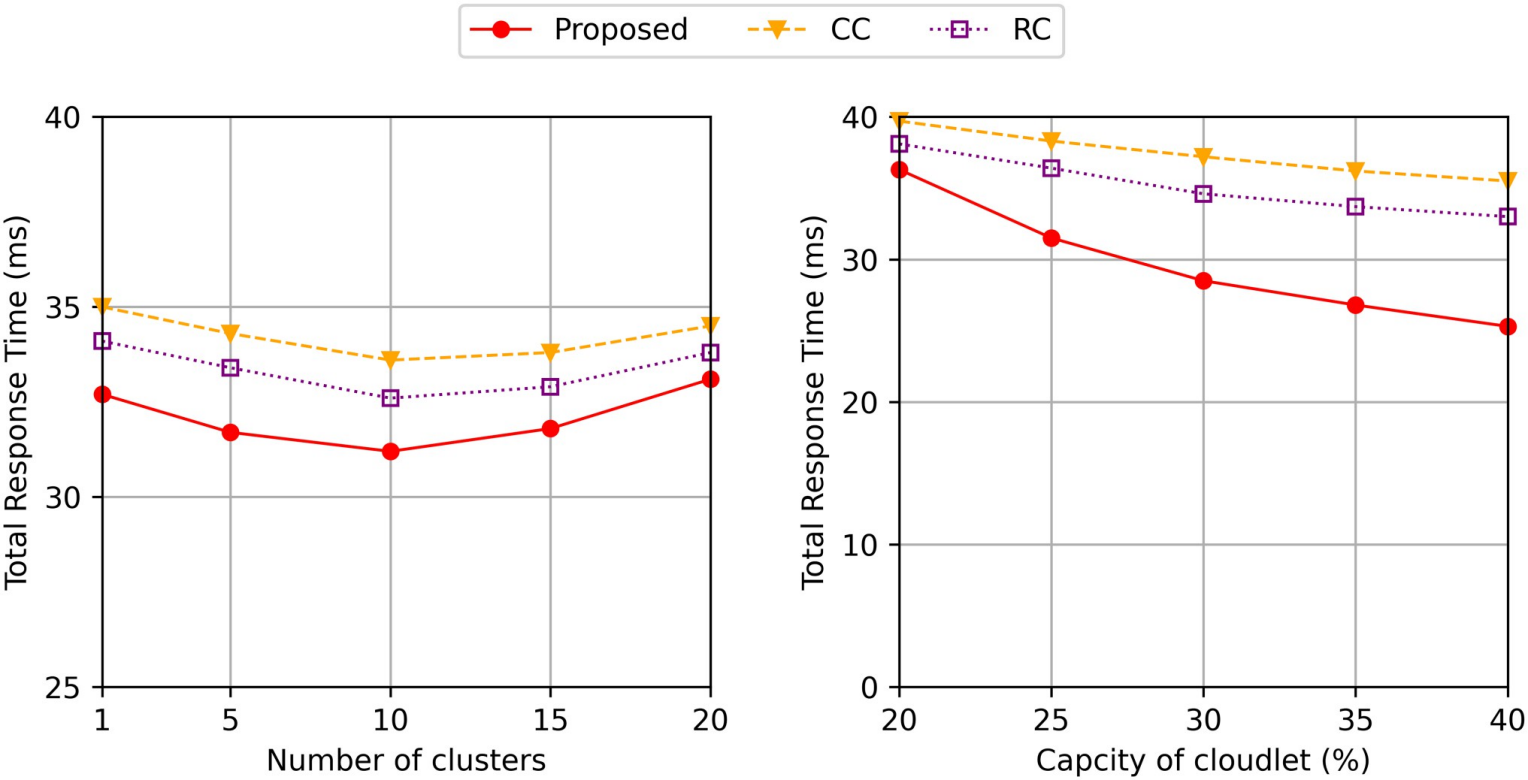
Total response time varying (left) the number of clusters and (right) the caching capacity of the cloudlet.

Fig 7 (Left) shows the total response time with the various number of clusters when the number of cloudlets is 100. The total response time decreases when the number of clusters increases from 1 to 10. When there are a fewer number of clusters, the size of each cluster gets bigger than when more number of clusters are formed. Hence, when there is one cluster, the task request is sent to the further cloudlet leading to a longer transmission time than when 5 and 10 clusters are formed. Interestingly, the total response time increases when the number of clusters increases from 10 to 20. Although the request is sent to the further cloudlet for the case of 10 clusters than for the case of 20 clusters, the difference in the number of cloudlets is less than when the number of clusters is increased from 1 to 10, as shown in Table 1. There are 10 cloudlets with the average of 1.34 replicas in each cloudlet when 10 clusters are formed, whereas only 5 cloudlets with an average of 1.08 replicas when 20 clusters are formed. Therefore, the task requests can be executed in a fewer number of cloudlets for the case of 20 clusters, compared to the case of 10 clusters, leading to a long waiting time. The proposed algorithm improves the total response time up to 7% and 4% than CC and RC, respectively.

**Table 1:** The maximum number of cloudlets, the maximum and average number of replicas in each cluster for different number of clusters.

Number of clusters	1	5	10	15	20
Max. number of cloudlets	100	20	10	6	5
Max. number of replicas	8	6	5	4	3
Avg. number of replicas	2.12	1.78	1.34	1.16	1.08

Fig 7 (Right) shows the total response time with the various caching capacity of the cloudlet. As shown in Fig 7 (right), the difference in total response time between the proposed algorithm and the two comparing methods gets larger when the capacity increases. Since the proposed algorithm caches the replicas of the popular data, the total response time increases when the storage capacity of the cloudlet decreases. However, the other two comparing methods do not overcome the proposed method even if the storage capacity of the cloudlet is less than 20% of the total size of unique program data in the system.

## 7 Conclusion & future work

In this paper, we have considered the cached-enabled IoT-edge system architecture, where the cloudlet can cache the program data for executing the task from IoT users. Caching the program data effectively reduces the total response time caused by loading the necessary program for the task. Considering the limited computation capacity and the storage of the cloudlets, we studied the cooperative caching architecture to handle the numerous task request from the IoT users. We then formulate the caching problem with the objective to minimize the total response time. For the problem formulation, we model the waiting time of task requests at the edge cloudlet as an M/G/1 queuing system. Based on the proposed architecture, we suggest the caching algorithm that caches the replicas of the popular program data into multiple cloudlets to reduce the total response time. Extensive simulation shows that the proposed caching algorithm with the clustering process achieves a lower response time compared to tje other two existing methods. Finally, we conclude the paper with some future working directions for the program caching in the IoT-Edge system. It is interesting to consider the task with various types of applications that have different deadlines. We also can consider using the machine learning method to predict the popularity of the program data in the region.

## Supporting information

S1 File (PDF)

## References

[1] Alnoman A., Sharma S. K., Ejaz W. and Anpalagan A. Emerging edge computing technologies for distributed IoT systems. IEEE Network, vol. 33, no. 6, pp. 140–147, 2019. doi: 10.1109/MNET.2019.1800543

[2] Yang Y., Zheng X., Guo W., Liu X., Chang V. Privacy-preserving smart IoT-based healthcare big data storage and self-adaptive access control system. Information Science, vol 479, pg. 567–592, 2019. doi: 10.1016/j.ins.2018.02.005

[3] Fazio M., Ranjan R., Girolami M., Taheri J., Dustdar S. and Villari M. A note on the convergence of IoT, edge, and cloud computing in smart cities. IEEE Cloud Computing, vol. 5, no. 5, pp. 22–24, 2018. doi: 10.1109/MCC.2018.053711663

[4] Yao J. and Ansari N. Caching in dynamic IoT networks by deep reinforcement learning. IEEE Internet of Things Journal, vol. 8, no. 5, pp. 3268–3275, 2021. doi: 10.1109/JIOT.2020.3004394

[5] E. Jonas, J. Smith, V. Sreekan, C. Tsai, A. Khan, Q. Pu, et al. Cloud programming simplified: a Berkeley view on serverless computing. arXiv:1902.03383 [Preprint], 2019.

[6] Bi S., Huang L. and Zhang Y. A. Joint optimization of service caching placement and computation offloading in mobile edge computing systems. IEEE Transactions on Wireless Communications, vol. 19, no. 7, pp. 4947–4963, 2020. doi: 10.1109/TWC.2020.2988386

[7] Z. Zhang, C. -H. Lung, I. Lambadaris and M. St-Hilaire. IoT data lifetime-based cooperative caching scheme for ICN-IoT networks. IEEE International Conference on Communications (ICC), pp. 1–7, 2018.

[8] S. Zhang, W. Sun and J. Liu. An optimized spatially cooperative caching strategy for heterogeneous caching network. 15th International Wireless Communications and Mobile Computing Conference (IWCMC), pp. 1685–1689, 2019.

[9] Piao Z., Peng M., Liu Y. and Daneshmand M. Recent advances of edge cache in radio access networks for internet of things: techniques, performances, and challenges. IEEE Internet of Things Journal, vol. 6, no. 1, pp. 1010–1028, 2019. doi: 10.1109/JIOT.2018.2866709

[10] Sun X. and Ansari N. Dynamic resource caching in the IoT application layer for smart cities. IEEE Internet of Things Journal, vol. 5, no. 2, pp. 606–613, 2018. doi: 10.1109/JIOT.2017.2764418

[11] Liu Y., Peng M., Shou G., Chen Y. and Chen S. Toward edge intelligence: multiaccess edge computing for 5G and internet of things. IEEE Internet of Things Journal, vol. 7, no. 8, pp. 6722–6747, 2020. doi: 10.1109/JIOT.2020.3004500

[12] Xu X., Feng C., Shan S., Zhang T. and Loo J. Proactive edge caching in content-centric networks with massive dynamic content requests. IEEE Access, vol. 8, pp. 59906–59921, 2020. doi: 10.1109/ACCESS.2020.2983068

[13] Fang J. and Ma A. IoT application modules placement and dynamic task processing in edge-cloud computing. IEEE Internet of Things Journal, vol. 8, no. 16, pp. 12771–12781, 2021. doi: 10.1109/JIOT.2020.3007751

[14] Q. Xie, Q. Wang, N. Yu, H. Huang and X. Jia. Dynamic service caching in mobile edge networks. IEEE International Conference on Mobile Ad Hoc and Sensor Systems (MASS), pp. 73–79, 2018.

[15] Zhang S., Guo S., Yu H., Wang Q. Cooperative service caching and computation offloading in multi-access edge computing. Computer Networks, vol. 189, pp. 107916, 2021. doi: 10.1016/j.comnet.2021.107916

[16] Velasquez K., Abreu D. P., Curado M. and Monteiro E. Service placement for latency reduction in the fog using application profiles. IEEE Access, vol. 9, pp. 80821–80834, 2021. doi: 10.1109/ACCESS.2021.3085370

[17] Li L. and Zhang H. Delay optimization strategy for service cache and task offloading in three-tier architecture mobile edge computing system. IEEE Access, vol. 8, pp. 170211–170224, 2020. doi: 10.1109/ACCESS.2020.3023771

[18] Kes M., Rez M., Sep A. Delay-aware optimization of energy consumption for task offloading in fog environments using metaheuristic algorithms. Cluster Computing, vol. 24, pp. 1825–1853, 2021. doi: 10.1007/s10586-020-03230-y

[19] Hao Y., Miao Y., Hu L., Hossain M. S., Muhammad G. Smart edge CoCaCo: AI enabled smart edge with joint computation, caching, and communication in heterogeneous IoT. IEEE Network, vol. 33, no. 2, pp. 58–64, 2019. doi: 10.1109/MNET.2019.1800235

[20] Liu P., Xu G., Yang K., Wang K. and Meng X. Jointly optimized energy-minimal resource allocation in cache-enhanced mobile edge computing systems. in IEEE Access, vol. 7, pp. 3336–3347, 2019. doi: 10.1109/ACCESS.2018.2889815

[21] Li Q., Zhong J., Cao Z. and Li X. Optimizing streaming graph partitioning via a heuristic greedy method and caching strategy. Optimization Methods and Software, vol. 35, pp. 1144–1159, 2020. doi: 10.1080/10556788.2018.1553971

[22] Duan P., Jia Y., Liang L., Rodriguez J., Huq K. M. S. and Li G. Space-reserved cooperative caching in 5G heterogeneous networks for industrial IoT. IEEE Transactions on Industrial Informatics, vol. 14, no. 6, pp. 2715–2724. 2018. doi: 10.1109/TII.2018.2794615

[23] B. Gao, Z. Zhou, F. Liu and F. Xu. Winning at the starting line: joint network selection and service placement for mobile edge computing. IEEE International Conference on Computing Communications, pp. 1459–1467, 2019.

[24] Z. Xu, L. Zhou, S. Chi-Kin Chau, W. Liang, Q. Xia and P. Zhou. Collaborate or separate? distributed service caching in mobile edge clouds. IEEE Conference on Computer Communications, pp. 2066–2075, 2020.

[25] Zhang C., Zhao H. and Deng S. A density-based offloading strategy for IoT devices in edge computing systems. IEEE Access, vol. 6, pp. 73520–73530, 2018. doi: 10.1109/ACCESS.2018.2882452

[26] Li C. and Jing Z. Dynamic cooperative caching strategy for delay-sensitive applications in edge computing environment. The Journal of Supercomputing, vol. 76, pp. 7594–7618, 2020. doi: 10.1007/s11227-020-03191-4

[27] Yousefpour A., Ishigaki G., Gour R. and Jue J. P. On reducing IoT service delay via fog offloading. IEEE Internet of Things Journal, vol. 5, no. 2, pp. 998–1010, 2018. doi: 10.1109/JIOT.2017.2788802

[28] Zhu X. and Zhou M. Multiobjective optimized cloudlet deployment and task offloading for mobile-edge computing. IEEE Internet of Things Journal, vol. 8, no. 20, pp. 15582–15595, 2021. doi: 10.1109/JIOT.2021.3073113

[29] Zhang S., He P., Suto K., Yang P., Zhao L. and Shen X. Cooperative edge caching in user-centric clustered mobile networks. IEEE Transactions on Mobile Computing, vol. 17, no. 8, pp. 1791–1805, 2018. doi: 10.1109/TMC.2017.2780834

[30] Abkenar F. S., Khan K. S. and Jamalipour A. Smart cluster-based distributed caching for fog-IoT networks. IEEE Internet of Things Journal, vol. 8, no. 5, pp. 3875–3884, 2020. doi: 10.1109/JIOT.2020.3026322

[31] Wang S., Li Q., Hou J., Meng S., Zhang B. and Zhou C. Active defense by mimic association transmission in edge computing. Mobile Networks and Applications, vol. 25, pp. 725–742, 2020. doi: 10.1007/s11036-019-01446-w

[32] Lai S., Fan X., Ye Q., Tan Z., Zhang Y., He X. et al. FairEdge: A fairness-oriented task offloading scheme for Iot applications in mobile cloudlet networks. IEEE Access, vol. 8, pp. 13516–13526, 2020. doi: 10.1109/ACCESS.2020.2965562

[33] Babar M., Khan M. S., Ali F., Imran M. and Shoaib M. Cloudlet computing: recent advances, taxonomy, and challenges. IEEE Access, vol. 9, pp. 29609–29622, 2021.

[34] Yao J. and Ansari N. Joint content placement and storage allocation in C-RANs for IoT sensing service. IEEE Internet of Things Journal, vol. 6, no. 1, pp. 1060–1067, 2019. doi: 10.1109/JIOT.2018.2866947

[35] Mukherjee A., De D. and Roy D. G. A power and latency aware cloudlet selection strategy for multi-cloudlet enviornment. IEEE Transactions on Cloud Computing, vol. 7, no. 1, pp. 141–154, 2019. doi: 10.1109/TCC.2016.2586061

[36] Wei H., Luo H., Sun Y. and Obaidat M. S. Cache-aware computation offloading in IoT systems. IEEE Systems Journal, vol. 14, no. 1, pp. 61–72, 2019. doi: 10.1109/JSYST.2019.2903293

[37] Chen G., Wu J., Yang W., Bashir A. K., Li G. and Hammoudeh M. Leveraging graph convolutional-LSTM for energy-efficient caching in blockchain-based green IoT. IEEE Transactions on Green Communications and Networking, vol. 5, no. 3, pp. 1154–1164, 2021. doi: 10.1109/TGCN.2021.3069395

[38] Lin P., Khan K. S., Song Q. and Jamalipour A. Caching in heterogeneous ultradense 5G networks. IEEE Vehicular technology magazine, vol. 14, no. 2, pp. 22–32, 2019. doi: 10.1109/MVT.2019.2904748

[39] M. Amadeo, G. Ruggeri, C. Campolo, A. Molinaro and G. Mangiullo. Caching popular and fresh IoT contents at the edge via named data networking. IEEE Conference on Computer Communications Workshops, pp. 610–615, 2020.

[40] Samanta A., Esposito F. and Nguyen T. G. Fault-tolerant mechanism for edge-based IoT networks with demand uncertainty. IEEE Internet of Things Journal, vol. 8, no. 23, pp. 16963–16971, 2021. doi: 10.1109/JIOT.2021.3075681

[41] Bhilwar H., Ranga V. and Gargi A. A critical power analysis for control path of a CAT-M based edge device. International Journal of Information Technology, vol. 13, pp. 845–855, 2021. doi: 10.1007/s41870-021-00640-y

[42] Yan J., Bi S. and Zhang Y. J. A. Offloading and resource allocation with general task graph in mobile edge computing: a deep reinforcement learning approach. IEEE Transactions on Wireless Communications, vol. 19, no. 8, pp. 5404–5419, 2020. doi: 10.1109/TWC.2019.2943563

[43] Peng K., Nie J., Kumar N., Cai C., Kang J., Xiong Z., et al. Joint optimization of service chain caching and task offloading in mobile edge computing. Applied Soft Computing Journal, vol. 103, pp. 107–142, 2021. doi: 10.1016/j.asoc.2021.107142

