# Supplementary material for: Delay-aware distributed program caching for IoT-edge networks
Source: PLoS One. 2022 Jul 19;17(7):e0270183. doi: 10.1371/journal.pone.0270183 (PMC9295985; doi:10.1371/journal.pone.0270183)
Supplement: S1 File — (PDF) [file pone.0270183.s001.pdf]

Figure	Subfigure	Data Points									
Figure2		31.871	32.232	32.613	33.026	33.546	34.057	34.523	34.91	35.612	36.3
	a	32.582	32.917	33.484	34.04	34.572	35.203	35.742	36.29	37.217	37.998
		31.925	32.57	33.018	33.534	33.841	34.375	34.768	35.277	35.963	36.609
		32.86	33.097	33.508	33.974	34.271	34.73	35.434	36.229	36.935	37.99
	b	33.4886	33.92	34.41848	34.907	35.43	36.36	37.463	38.613	40.088	42.205
		33.125	33.425	33.914	34.322	34.7	35.41	36.116	36.742	37.836	38.788
		33.463	33.59	33.771	33.987	34.256	34.639	35.123	35.814	36.551	37.267
	c	33.757	34.102	34.399	34.709	35.2	35.9	36.925	38.335	40.433	43.779
		33.549	33.766	34.02	34.338	34.721	35.21	35.679	37.016	38.164	40.386
Figure3		26.38112	26.80592	27.29296	28.129	28.596	29.3	30.003	30.742	32.453	34.149
	a	29.6	30.249	31.014	31.902	33.166	34.914	36.793	40.12	43.697	62.953
		29.143	29.672	30.546	31.369	32.554	34.177	35.62	38.205	41.362	53.623
		27.292	27.5144	27.76832	27.91	28.169	28.445	28.695	28.903	29.201	29.634
	b	28.251	28.387	28.796	29.263	29.782	31.146	33.527	36.209	46.8	59.659
		27.72	27.864	28.249	28.746	29.299	30.603	31.798	34.698	41.62	51.803
Figure4		13.305	13.3231	13.353	13.428	13.493	13.551	13.656	13.742	13.822	13.955
	a	14.234	14.423	14.773	1.115	15.536	16.293	17.189	19.088	20.931	23.531
		14.053	14.152	14.323	14.703	14.972	15.431	16.102	17.823	19.742	22.321
		35.434	36.286	37.847	39.433	42.174	49.195	60.655	70.576	80.562	97.233
	b	40.565	42.736	47.147	53.645	64.181	77.714	106.752	131.645	180.132	229.754
		37.274	40.432	45.642	50.545	58.343	65.844	90.345	114.546	162.523	208.543
Figure5		16.48	33.99	55.62	78.28	105.06					
	a	18.54	40.17	65.199	101	133.9					
		17.51	36.05	56.65	83.7	117.815					
		2.6677	1.9252	3.296	3.934	4.439	5.256	6.077	6.981	7.622	8.537
	b	5.9431	6.9731	7.925	9.62	11.433	14.008	16.583	18.96	21.526	24.72
		3.141	3.296	3.605	4.65	5.665	6.901	8.31	9.396	10.668	11.948
		152.35	107.234	61.23	34.8	32.9	32.3	31.9			

	a	190.7	156.3	110.8	70.7	55.3	52.2	50.2
		173.2	128.2	82.3	54.2	38.5	36	35.4
		147.25	97.72	57.13	38.3	37.7	36.3	35.2
Figure6	b	218.7	167.3	130.3	110.2	97.3	87.1	76.4
		201.1	142.1	106.8	78.78	70.7	65.2	60.5
		17.25	97.72	57.13	38.3	37.7	36.3	35.2
	c	218.7	167.3	130.3	110.2	97.3	87.1	76.4
		201.1	142.1	106.8	78.78	70.7	65.2	60.5
		32.7	31.7	31.2	31.8	33.1		
	a	35	34.3	33.6	33.8	34.5		
		34.1	33.4	32.6	32.9	33.8		
Figure7		36.3	31.5	28.5	26.8	25.3		
	b	39.7	38.3	37.2	36.2	35.5		
		38.1	36.4	34.6	33.7	33		
